# The Immunogenicity of CpG, MF59-like, and Alum Adjuvant Delta Strain Inactivated SARS-CoV-2 Vaccines in Mice

**DOI:** 10.3390/vaccines12010060

**Published:** 2024-01-07

**Authors:** Kangwei Xu, Jing Li, Xu Lu, Xiaoqin Ge, Kaiqin Wang, Jiahao Wang, Zhizhong Qiao, Yaru Quan, Changgui Li

**Affiliations:** 1 National Institutes for Food and Drug Control, NHC Key Laboratory of Research on Quality and Standardization of Biotech Products, NMPA Key Laboratory for Quality Research and Evaluation of Biological Products, No. 2, Tiantan Xili, Dongcheng District, Beijing 100050, China; xukw@nifdc.org.cn (K.X.);; 2 Sinovac Life Sciences Co., Ltd., No. 21, Tianfu St., Daxing Biomedicine Industrial Base of Zhongguancun Science Park, Daxing District, Beijing 100050, China

**Keywords:** SARS-CoV-2, inactivated vaccines, Delta, immunogenicity, adjuvants

## Abstract

The continuous evolution and mutation of SARS-CoV-2 have highlighted the need for more effective vaccines. In this study, CpG, MF59-like, and Alum adjuvant Delta strain inactivated SARS-CoV-2 vaccines were prepared, and the immunogenicity of these vaccines in mice was evaluated. The Delta + MF59-like vaccine group produced the highest levels of S- and RBD-binding antibodies and live Delta virus neutralization levels after one shot of immunization, while mice in the Delta + Alum vaccine group had the highest levels of these antibodies after two doses, and the Delta + MF59-like and Delta + Alum vaccine groups produced high levels of cross-neutralization antibodies against prototype, Beta, and Gamma strain SARS-CoV-2 viruses. There was no significant decrease in neutralizing antibody levels in any vaccine group during the observation period. CpG, MF59-like, and Alum adjuvant Delta strain inactivated SARS-CoV-2 vaccines excited different antibody subtypes compared with unadjuvanted vaccines; the Delta + CpG vaccine group had a higher proportion of IgG2b antibodies, indicating bias towards Th1 immunity. The proportions of IgG1 and IgG2b in the Delta + MF59-like vaccine group were similar to those of the unadjuvanted vaccine. However, the Delta + Alum vaccine group had a higher proportion of IgG1 antibodies, indicating bias towards Th2 immunity. Antigen-specific cytokine secretion CD4/8^+^ T cells were analyzed. In conclusion, the results of this study show differences in the immune efficacy of CpG, MF59-like, and Alum adjuvant Delta strain inactivated SARS-CoV-2 vaccines in mice, which have significant implications for the selection strategy for vaccine adjuvants.

## 1. Introduction

Severe Acute Respiratory Syndrome Coronavirus 2 (SARS-CoV-2), the causative agent of the disease COVID-19, was first identified in human populations in 2019 and rapidly escalated into a global pandemic. The disease manifests in a spectrum of severity in patients, with most individuals experiencing mild symptoms and recovering within a few weeks. However, some develop serious complications such as pneumonia or acute respiratory distress syndrome which, unfortunately, have resulted in fatalities in some instances [1,2,3]. SARS-CoV-2 is categorized as an enveloped single-stranded RNA virus, falling under the genus Beta coronavirus within the Coronavirus family. It produces four structural proteins: nucleocapsid (N), glycoprotein (S), membrane (M), and envelope (E) proteins [4,5]. The S protein is composed of two subunits, S1 and S2. The receptor-binding domain (RBD) situated on the S1 subunits attaches to the angiotensin-converting enzyme 2 (ACE2) receptor in the host, while the S2 subunits facilitate the virus penetration into the host cells. Therefore, despite the N protein being the most abundant, the S protein is the primary target for the action of neutralizing antibodies [6,7,8,9].

In response to the urgent public health crisis presented by the COVID-19 pandemic, there has been a concerted global effort to expedite research and development processes for a vaccine. This international cooperation has yielded several types of vaccines that have undergone rigorous clinical trials and have subsequently been approved for use. The World Health Organization has sanctioned the emergency use of four primary types of vaccines: inactivated virus, RNA, non-replicating viral vector, and protein subunit vaccines [10]. Among these, inactivated vaccines, which contain a virus that has been rendered non-pathogenic, have a long history of use and are considered among the safest and most commonly utilized types of vaccines. These vaccines, distributed in billions of doses, have demonstrated their effectiveness in safeguarding against a wide variety of viral and bacterial diseases.

COVID-19 inactivated vaccines such as CoronaVac, BBIBP-CorV, and Covaxin have been widely used. These vaccines are prepared by inactivating cell culture viruses using chemical reagents such as β-propiolactone [11]. The vaccine maintains the integrity of the virus particle test and uses the whole virus as an immunogen. Compared with subunit vaccines of S protein or RBS fragments, a broader immune response can be induced [12,13]. Clinical trial results have shown that the overall adverse effects of inactivated vaccines are low, indicating a good safety profile [14,15,16,17]. They also have good safety among different populations, such as children and adolescents, the elderly, cancer patients, pregnant women, immunocompromised people, those with comorbidities, and HIV-infected people [18].

Adjuvants are often added to vaccines to improve the efficiency of the immune response, increase the duration of protection, reduce the amount of antigen used, and provide cross-protection by enhancing the breadth of the immune response to multiple antigens. At present, more than 10 adjuvants have been used in human vaccines, including aluminum salt, MF59, CpG, AS01, AS03, AS04, liposomes, etc. Aluminum salts are the most widely used adjuvant, used in billions of vaccine doses [19,20]. The inactivated vaccines CoronaVac and BBIBP-CorV both use aluminum hydroxide as adjuvants, while Covaxin uses aluminum hydroxide with toll-like receptor 7/8 agonist molecule (IMDG) as adjuvants [11,21]. In this study, the Delta strain virus was purified and inactivated after in vitro culture, and combined with an aluminum adjuvant, a MF59-like adjuvant, or a CpG adjuvant, and the immune effects of the different adjuvant inactivated vaccines were evaluated in mice.

## 2. Materials and Methods

### 2.1. Preparation of Inactivated Delta Vaccine with Different Adjuvants

The Delta (B.1.617.2) strain virus of SARS-CoV-2 was isolated from a COVID-19 patient and an adaptive subculture on Vero cells was cultured. Preparation of the inactivated Delta vaccine bulk was consistent with previous reports [9,11]. Briefly, the Delta strain virus was propagated in Vero cells (Obtained from WHO) for large-scale culture, and β propiolactone was added to the culture product at a ratio of 1:4000 for virus inactivation. After purification via ultrafiltration, ion exchange, molecular sieving, and other purification steps, the inactivated Delta vaccine bulk was obtained. Delta vaccines were prepared by combining the inactivated Delta vaccine bulk with different adjuvants. A total of five groups of vaccines and a control were prepared in this study (Table 1). The choice of an inactivated Delta virus and aluminum adjuvant immunization dose was based on our previous study [11], and the CpG and MF59-like adjuvants were used at the manufacturer’s recommended dose. The Delta vaccine was prepared by diluting inactivated Delta vaccine bulk to 6 μg/mL of protein using PBS. The Delta + CpG vaccine was prepared by diluting inactivated Delta vaccine bulk to 12 μg/mL of protein using PBS and mixed with CpG adjuvants (Synbio Tech, Chengdu, China) with an equal volume concentration of 40 μg/mL. The Delta + MF59-like vaccine was prepared by diluting inactivated Delta vaccine bulk to 12 μg/mL of protein using PBS and then mixed with an equal volume of MF59-like adjuvant (InvivoGen, Toulouse, French) before use. The Delta + Alum vaccine was prepared by diluting inactivated Delta vaccine bulk to 12 μg/mL protein content using PBS and mixed with aluminum hydroxide adjuvant (SINOVAC, Beijing, China) with an equal volume concentration of 1 mg/mL, letting it stand for 24 h. PBS was used as a negative control. Each dose of the vaccine preparation was 0.5 mL.

### 2.2. Mouse Immunization

**Figure 1:**
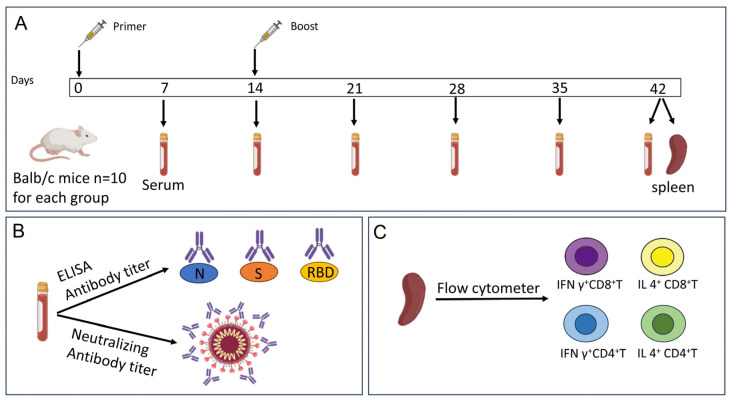
Animal immunization and sample analysis strategy. Balb/c mice were immunized at days 0 and 14. Serum was collected at 7, 14, 21, 28, 35, and 42 days, and spleens were isolated 42 days post-primer vaccination (**A**). Antibodies against S, RBD, N proteins, and neutralizing antibody in mouse serum were detected via indirect ELISA and microneutralization test methods (**B**). Spleens were analyzed using flow cytometry to measure the percentages of IFN γ+ and IL 4+ in CD4+/CD8+ T cells, respectively (**C**).

This study was approved by the Institutional Animal Care and Use Committee at the National Institutes for Food and Drug Control, China. Female SPF-level Balb/c mice, 6 weeks old, were selected as experimental animals, and 10 mice were immunized in each group. Immunization was performed twice via intraperitoneal injection of 0.5 mL of vaccine on days 0 and 14. Blood was collected and serum was isolated 7, 14, 21, 28, 35, and 42 days after the first immunization; ELISA and neutralizing antibody tests were performed. The mouse spleen was removed at 42 days for flow cytometry assay (Figure 1).

### 2.3. Indirect ELISA Assay

The indirect ELISA method was used to detect antibodies against S, RBD, and N proteins in mouse serum. The recombinant Delta strain SARS-CoV-2 N, S, or RBD proteins (SinoBiological, Beijing, China, N Cat: 40588-V07E29, S Cat: 40589-V08B16, RBD Cat: 40592-V08H90) were diluted to 1 μg/mL using PBS. Ninety-six-well ELISA plates were coated with antigens at 100 μL/well and placed in 4 °C refrigeration overnight. After washing the plate with PBS-T solution five times, 200 μL/well blocking solution (1% BSA in PBS) was added and blocking was undertaken for 1 h at room temperature. After discarding the blocking solution, mouse serum diluted with blocking solution was added, with 2-fold dilution starting at 800-fold, and samples were incubated at 37 °C for 1 h. After washing five times with PBS-T solution, HRP-labeled anti-mouse IgG antibody (Seracare/KPL, Milford, MA, USA) was added and diluted 10,000-fold with blocking solution. This was followed by washing the plate five more times. TMB substrate (Seracare/KPL, Milford, MA, USA) was added to the plate to produce a color change, and A450/630 was measured using a plate reader. Serum titer was defined as a dilution factor greater than 2.1 times the absorbance value of the negative well. An antibody titer less than 800 was assigned a value of 400 for the GMT calculation. For serum samples 42 days after immunization, tests for IgG antibody subtypes were also performed. The above steps were used for S protein coating and serum sample dilution, and HRP-labeled anti-mouse IgG antibodies were replaced with HRP-labeled anti-mouse IgG1/2a/2b/3 (Southern Biotech, Oxmoor Blvd, Birmingham, AL, USA) antibodies to detect the titer of different isotypes of antibodies.

### 2.4. Neutralizing Antibody Detection

Neutralizing antibody titers in mouse serum were determined using microneutralization tests, which were conducted in a biosafety level 3 (BSL-3) laboratory. Serum samples underwent inactivation in a water bath set at 56 °C for 30 min, then were diluted eight times with 199 media. Subsequently, 50 μL of the sample was placed into each of two wells in a pre-filled cell culture plate, which was then subjected to a two-fold dilution. Using 199 media, the SARS-CoV-2 Delta strain virus was diluted to 100 CCID_50_/50 μL, and 50 μL was added per well to the cell culture plate for neutralization in a 37 °C, 5% CO_2_ incubator for a duration of 120 min. Vero cells were diluted to a concentration of 1.2–2.0 × 10^5^ cells/mL, and 100 μL was added to each well of the neutralized cell culture plate. The plate was then incubated for five days in a 37 °C, 5% CO_2_ incubator. Cytopathies were visually inspected under a microscope, and neutralizing antibody titers were calculated based on the dilution at which half the cytopathies were presented. When calculating the geometric mean titers (GMTs), any antibody titers under 8 were given a value of 4. Additionally, 42 days post-immunization, neutralizing titers were also conducted against the prototype, Beta, and Gamma strains of SARS-CoV-2 using serum samples.

### 2.5. Flow Cytometry Analysis

After 42 days from the first immunization, mouse spleens were removed for antigen-specific T cell testing. Mouse spleens were suspended in PBS for trituration into a homogenate, incubated with Lysing buffer to lyse red blood cells, and subsequently filtered through a 40 μm cell strainer to obtain single splenocytes. Approximately 1 million splenocytes were seeded into each well, followed by the addition of a SARS-CoV-2 S peptide pool (SinoBiological, Beijing, China) and cultured at 37 °C with 5% CO_2_ for 4 h. T cell surface markers were stained using anti-mouse CD3 (BV510), anti-mouse CD4 (FITC), anti-mouse CD8 (Brilliant Violet 785), and anti-mouse CD25 (Brilliant Violet 421). Following washing, cells were treated with Fixation and Permeabilization Solution. After incubation for 30 min at 4 °C in the dark and washing with Permeabilization washing buffer, anti-mouse IFN γ (PE) and anti-mouse IL4 (PE-CF594) were added for cytokine detection. The percentage of antigen-specific cytokine secretion T cells were analyzed using flow cytometry (NovoCyte D3130, AECA, Santa Clara, CA, USA).

### 2.6. Statistical Analyses

GraphPad Prism 8.0.1 was applied for statistical analysis. Binding antibodies against SARS-CoV-2 S, RBD, and N proteins and neutralization antibodies are presented as geometric mean titers (GMTs). Antigen-specific cytokine secretion T cells are presented as mean percentages. Significance was considered when *p* values were < 0.05.

## 3. Results

### 3.1. Detection of Antibodies against S, RBD, and N Proteins in Mouse Serum

**Figure 2:**
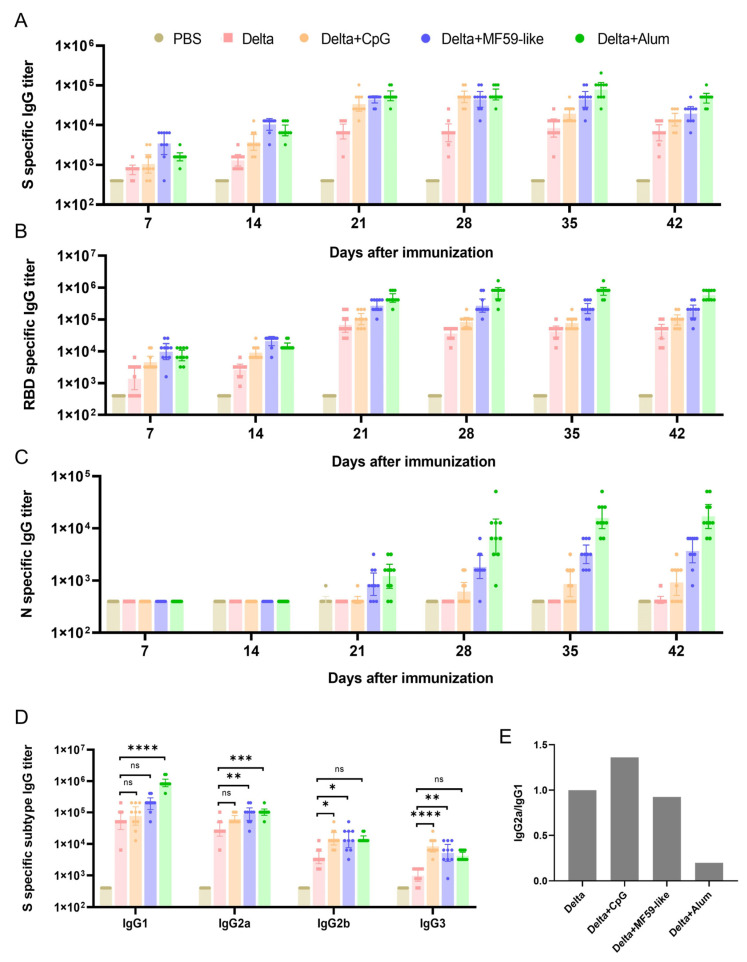
Serum binding antibody titers after immunization. Five groups of mice (*n* = 10) were immunized with Delta inactivated vaccine with different adjuvants at 14-day intervals. The changes in antibody titers against the S protein (**A**), RBD (**B**), and N protein (**C**) in serum at 7, 14, 21, 28, 35, and 42 days after the first immunization were measured. IgG isotype analysis against S protein was performed on serum 42 days after immunization, antibody titers against IgG1, IgG2a, IgG2b, and IgG3 were detected (**D**), and the relative values of IgG2a/IgG1 and the unadjuvanted Delta vaccine group were calculated (**E**). The data points represent the values detected from each mouse, the reference column height is the geometric mean of the titer (GMT), and the error bars indicate the 95% confidence interval. Statistical analyses were conducted using one-way ANOVA with Dunnett’s multiple comparisons test. ns, *p* > 0.05; *, *p* < 0.05; **, *p* < 0.01; ***, *p* < 0.001; ****, *p* < 0.0001.

Recombinant SARS-CoV-2 S, RBD, N proteins were used to detect the titer of bound antibodies in the serum of immunized mice. S protein antibodies were detected in mice with and without adjuvant vaccine groups seven days after the first immunization (Figure 2A). The 7-day and 14-day antibody titers of the Delta + MF59-like vaccine group were the highest, while the antibody titers in the Delta + CpG vaccine group were lower, which was similar to that in the unadjuvanted Delta vaccine group. After the booster immunization on the 14th day, the antibody titers of mice in all vaccine groups increased significantly, and the Delta + Alum vaccine group had the highest antibody titers. After the antibody titer in the Delta + CpG vaccine group reached the highest level on day 28, the antibody titer decreased significantly on day 35 and day 42. However, the antibody titers of the Delta + MF59-like and Delta + Alum vaccine groups decreased slightly after 35 days, suggesting that the two adjuvanted vaccines had better antibody persistence. The results of the RBD antibody test (Figure 2B) were similar to those of the S protein antibody, with the highest antibody titers in the Delta + MF59-like vaccine group at 7 and 14 days after the initial immunization, and the highest antibody titers in the Delta + Alum vaccine group after booster immunization. Unlike S protein antibodies, RBD-specific antibodies had better persistence, and there was no significant decrease in RBD antibody titers in each vaccine group during the 42-day observation period. At 7 and 14 days after the initial immunization, none of the groups exhibited N-specific antibodies (Figure 2C), and after booster immunization, the Delta + Alum vaccine group had the highest antibody titers, while the unadjuvanted Delta vaccine group still did not produce N-specific antibodies. This indicates that the Delta strain SARS-CoV-2 virus has poor N-protein immunogenicity, which is also consistent with our previous research results on the prototype virus [11].

### 3.2. Detection of IgG Subtype Antibodies against S Proteins in Mouse Serum

Next, we performed a titer analysis of the IgG subtype in serum collected over 42 days (Figure 2D). The IgG1 subtype titer was the highest in each group. Compared with the unadjuvanted Delta vaccine group, the enhancement of antibody subtypes was different between the three adjuvant vaccine groups: the antibody titers of IgG2b and IgG3 subtypes were significantly increased in the Delta + CpG vaccine group, the IgG2a, IgG2b, and IgG3 subtype antibody titers were significantly increased in the Delta + MF59-like vaccine group, and the IgG1 and IgG2a subtype antibody titers were significantly increased in the Delta + Alum vaccine group. The ratio of IgG2a/IgG1 antibody titers to the unadjuvanted Delta vaccine group was calculated, and the results are shown in Figure 2E. The Delta + CpG vaccine group is biased to IgG2a antibody response (Th1), the Delta + MF59-like vaccine group has a similar antibody response bias to the unadjuvanted Delta vaccine group, and the Delta + Alum vaccine group is more biased towards IgG1 antibody response (Th2).

### 3.3. Neutralizing Antibody Titers

**Figure 3:**
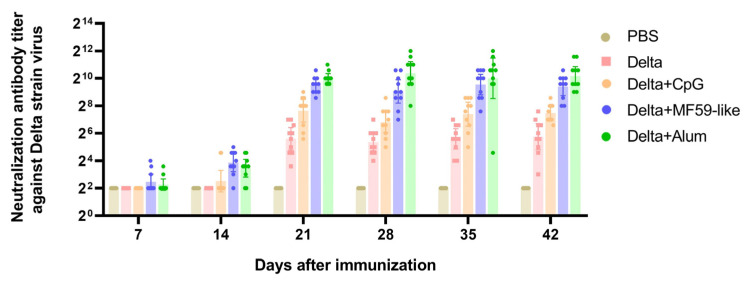
The serum neutralization titer was detected using a micro-neutralization assay. The live virus of the Delta strain was used for micro-neutralization experiments, and the serum neutralizing antibody titers of mice 7, 14, 21, 28, 35, and 42 days after the first immunization of each group were detected. The data points represent the values detected from each mouse, the reference column height is the geometric mean of the titer (GMT), and the error bars indicate the 95% confidence interval.

The micro-neutralization method was used to detect the titer of Delta virus neutralizing antibody in the serum of immunized mice. As shown in Figure 3, neutralizing antibodies were detected in some mice in the Delta + MF59-like and Delta + Alum vaccine groups seven days after the first immunization. By day 14, neutralizing antibodies were detectable in most mice in the two groups, and the neutralizing antibody levels were slightly higher in the Delta + MF59-like vaccine group. After the booster immunization on day 14, neutralizing antibodies were detectable in mice in all vaccine groups. The neutralizing antibody levels in the Delta + MF59-like and Delta + Alum vaccine groups were higher than those in the unadjuvanted Delta and Delta + CpG vaccine groups, and the neutralizing antibody titers in the Delta + Alum vaccine group were the highest. During the 42-day observation period, there was no significant decrease in neutralizing antibody titers in each vaccine group. These results are consistent with RBD-binding antibody test results.

Next, we measured serum neutralizing antibody levels against the prototype, Beta, and Gamma strains of the SARS-CoV-2 virus at 42 days post-immunization (Table 2). The Delta + Alum vaccine group with the highest neutralizing antibody titer against the Delta strain also had the highest neutralizing antibody level against the other three strains. The Delta + MF59-like vaccine group had a slightly lower neutralizing antibody level, and the Delta + CpG vaccine group had the lowest neutralizing antibody level among the three adjuvanted vaccine groups, but it was still higher than that of the unadjuvanted Delta vaccine group.

### 3.4. T Cell Immune Responses Elicited by Inactivated Delta Vaccines with Different Adjuvants

**Figure 4:**
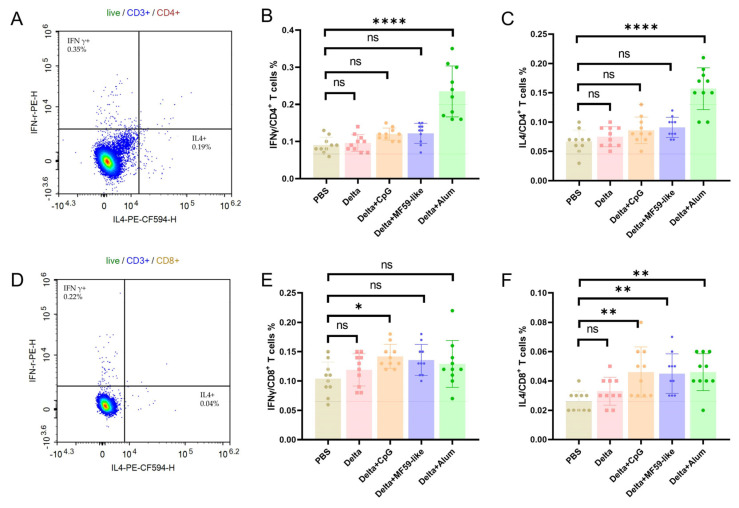
T cell immune responses in the vaccinated BALB/c mouse spleens. BALB/c mice received two doses of the inactivated Delta vaccine with various adjuvants at 14-day intervals. Splenocytes, isolated 42 days post-initial vaccination and stimulated with a SARS-CoV-2 S peptide pool, were analyzed using flow cytometry. The gating strategy of IFNγ/IL4 positive cells in CD3+CD4+/CD3+CD8+ T cells are presented in panel (**A**) and (**D**). The results of percentages of IFN γ+ in CD4+ T cells (**B**); IL 4+ in CD4+ T cells (**C**); IFN γ+ in CD8+ T cells (**E**) and IL 4+ in CD8+ T cells (**F**), respectively. Data are presented as mean ± SEM (*n* = 10). Statistical analyses were conducted using one−way ANOVA with Dunnett’s multiple comparisons test. ns, *p* > 0.05; *, *p* < 0.05; **, *p* < 0.01; ****, *p* < 0.0001.

Two doses of inactivated Delta vaccine with different adjuvants were administered to BALB/c mice at 14-day intervals. At 42 days following the initial vaccination, mice were euthanized and splenocytes were isolated. The isolated splenocytes were then stimulated with a SARS-CoV-2 S peptide pool before the detection of T cell immune responses. Compared with the PBS group, a significant increase in the proportion of IFN γ+ CD4+ T and IL4+ CD4+ T cells was observed in mice from the Delta + Alum vaccine group. Slight increases in the proportions of IFN γ+ CD4+ T and IL4+ CD4+ T cells were noted in the Delta + CpG and Delta + MF59-like vaccine groups, although these changes were not statistically significant (Figure 4B,C). This trend correlates with the highest antibody titer levels observed at 42 days in mice from the Delta + Alum vaccine group. Increases in the proportions of IFN γ+ CD8+ T and IL4+ CD8+ T were observed for the Delta + CpG, Delta + MF59-like, and Delta + Alum vaccine groups compared with those from the PBS group, and the proportion of IFN γ+ CD8+ T cells in the Delta + CpG group was significantly increased (Figure 4E,F).

## 4. Discussion

Since the emergence of the COVID-19 pandemic, various vaccines including mRNA vaccines, adenovirus vector vaccines, recombinant vaccines, and inactivated vaccines have been utilized for the prevention and control of SARS-CoV-2, effectively reducing the rates of infection and severe cases. The mRNA vaccines, developed using new technology, were the first to be globally approved for the market. These vaccines were found to induce high titers of neutralizing antibodies and CD4/CD8 T cell responses in mice. In non-human primate models, mRNA-vaccinated groups exhibited significant viral clearance capabilities upon virus challenge [22,23]. Clinical studies indicate that mRNA vaccines have achieved over 90% efficacy in human populations [24,25]. Inactivated SARS-CoV-2 vaccines have also demonstrated protective effects in animal models [11]. Despite inducing lower levels of neutralizing antibodies in human populations and exhibiting lower efficiency in preventing symptomatic COVID-19 cases (ranging between 50.7% and 83.5%), they possess superior safety profiles [16,17,21,26]. Furthermore, the technology used to produce inactivated vaccines is mature and easily scalable for mass production. These vaccines can also be refrigerated and transported at temperatures of 2–8 °C without the need for freezing, providing significant advantages for global distribution, especially in developing countries. Thus, they remain a crucial tool against the pandemic threat of the virus [27].

The emergence of new COVID-19 variants has reduced the protective effects of various vaccines. The efficacy of mRNA vaccines against the Beta/Gamma variants ranges from 76 to 100%, against the Delta variant from 47.3 to 88%, and against the Omicron variant effectiveness has dropped to 23.7 from 47% [28,29,30,31,32,33]. The effectiveness of inactivated vaccines against the variants has also significantly decreased, with efficacy against the Delta variant ranging from 30.4 to 74.5% and against the Omicron variant at 37.9% [15,33,34]. The development of universal vaccines is an ideal approach to address emerging variants. Broad-spectrum vaccines prepared using different technologies have demonstrated protection against various VOCs in animal experiments, but no universal vaccines have been marketed so far [35,36,37,38]. Vaccines that include prevalent SARS-CoV-2 variants are a more feasible solution, and the WHO encourages the development of such vaccines on various platforms [39]. Vaccines with high immunogenicity that are capable of resisting SARS-CoV-2 variants can play a vital role in the prevention and control of the COVID-19 pandemic. This study involved preparing inactivated Delta variant vaccines containing three types of human adjuvants, CpG, MF59-like, and Alum, focusing on the immune response in mice and evaluating the role of adjuvants in order to screen for vaccines with better immunogenicity.

In this study, the effect of humoral immunity was first evaluated. After two immunizations on days 0 and 14, S- and RBD-specific IgG antibodies were rapidly produced in mice. The Delta + MF59-like vaccine group had the highest antibody titers on days 7 and 14 after the first immunization, suggesting that MF59-like can quickly induce antibody production. After the booster immunization on day 14, the Delta + Alum vaccine group had the highest antibody titers. The Delta + CpG group had the lowest titers among the three adjuvant vaccine groups, but these were still higher than the control group without an adjuvant. This is consistent with the results of Jie et al. for the prototype strain of the inactivated COVID-19 vaccine with different adjuvants [40]. As the most abundant protein in the SARS-CoV2 virus, N has an independent role in vaccine-induced protection [41]. Therefore, the N protein antibody was also evaluated in this study. After the second immunization, the three adjuvant vaccine groups produced low levels of N-protein-specific antibodies, suggesting that, like the prototype strain, the immunogenicity of the N protein of the Delta strain SARS-CoV-2 virus is also low [11]. The results of S-specific IgG antibody subtypes showed that regardless of the addition of adjuvants, the level of IgG1 antibodies was the highest. The titers of IgG1, IgG2a, IgG2b, and IgG3 subtype antibodies in the three adjuvant vaccine groups were all higher than those of the vaccine without adjuvants to varying degrees. Considering that the level of IgG1 antibodies is a marker of Th2 response, and IgG2a is a marker of Th1 response [42], we calculated the ratio of IgG2a/IgG1 of different vaccines and compared it with the group without adjuvants. The Delta + CpG group was more biased towards the Th1 response, the Delta + MF59-like vaccine group response was similar to the group without adjuvants, and the Delta + Alum vaccine group was more biased towards the Th2 response. Since balanced immune responses are more conducive to virus clearance, MF59-like adjuvant shows a promising ability to induce a more intense antiviral response in the host [43].

Preclinical studies in mice and non-human primates, as well as vaccine efficacy studies, suggest that neutralizing antibody levels are potential indicators for protection against SARS-CoV-2 [11,22,23]. In this study, after immunization in mice, the three adjuvant vaccine groups had higher levels of neutralizing antibodies than the group without an adjuvant, demonstrating an immunoenhancing effect. Similar to the results of S- and RBD-specific IgG antibody detection, the Delta + MF59-like vaccine group had the highest antibody titers on days 7 and 14 after the first immunization, and after the booster immunization on day 14, the Delta + Alum vaccine group had the highest antibody titers. Moreover, mice in the Delta + MF59-like and Delta + Alum vaccine groups not only produced high titers of neutralizing antibodies against the homologous Delta strain virus, but also showed good cross-neutralizing activity against the prototype, Beta, and Gamma strains of the SARS-CoV-2 virus, indicating their potential cross-protective effect.

Studies on patients infected with COVID-19 reveal that T-cell immunity is instrumental in combating SARS-CoV-2 infection. A decrease in T-cell counts has been linked to the emergence of severe conditions like pneumonia. On the other hand, adequate T-cell counts are associated with the rapid elimination of the virus and successful patient recovery [44,45]. Therefore, this study evaluated the S-protein-specific T cell immune response. Our results show that the antigen-specific CD4+ and CD8+ T cells in the three adjuvant vaccine groups were all higher than in the control group; in particular, the level of IFN γ+ CD8+ T cells in the Delta + CpG group was significantly increased. An advantage of the T-cell immune response is its ability to recognize conservative regions that exist in the S protein across various strains of the virus, suggesting that specific mutations of SARS-CoV-2 variants may not result in cellular immune evasion. Unlike COVID-19 vaccines targeting the S protein produced with other technologies, inactivated COVID-19 vaccines contain conservative antigens like the N protein. As antigenic changes in these conserved internal structural proteins are seldom observed in VOCs, T-cell immunity against these proteins is unlikely to be impacted by antibody escape mutations in VOCs. Therefore, inactivated COVID-19 vaccines are likely to remain effective against existing and future variants of SARS-CoV-2 [46,47,48].

There are some limitations in this study that need to be addressed. Firstly, although a large amount of previous research has shown a correlation between the level of neutralizing antibodies produced after vaccination and protective efficacy, this study did not conduct viral challenge research to directly evaluate the protective efficacy of different adjuvant vaccines. Secondly, due to some uncontrollable factors, we were unable to obtain mRNA vaccines for control experiments or the Omicron strain for cross-protection evaluation during the conduct of this study. This limits our ability to conduct a broader and deeper evaluation of cross-protection efficacy, which is crucial for assessing the effectiveness and efficiency of different vaccines against new virus variants. We are conducting research to address these issues.

As SARS-CoV-2 continues to mutate, the threat it poses in the future cannot be ignored. Our research has compared the immune effects of inactivated vaccines with different adjuvants in mice, providing a reference for the development of safer and more effective inactivated vaccines.

## Figures and Tables

**Table 1:** Vaccine formulations and study groups.

Vaccine	Antigen Content	Adjuvant
PBS	w/o	w/o
Delta	3 μg/dose	w/o
Delta + CpG	3 μg/dose	CpG 10 μg/dose
Delta + MF59-like	3 μg/dose	MF59-like 0.25 mL/dose
Delta + Alum	3 μg/dose	Aluminum hydroxide 250 μg/dose

**Table 2:** Neutralization antibodies to the Delta, prototype, Beta, and Gamma strains of the live SARS-CoV-2 virus (GMT and 95% confidence interval, *n* = 10 for each group).

	Virus	Delta	Prototype	Beta	Gamma
Vaccines					
Delta		53.85 (27.85~104.1)	26.61 (11.81~59.96)	7.87 (4.42~14.01)	6.06 (3.56~10.33)
Delta + CpG		178.0 (129.1~245.3)	56.08 (32.68~96.23)	22.9 (12.33~42.51)	22.25 (11.96~41.39)
Delta + MF59-like		672.1 (431.7~1046)	250.4 (125.3~500.3)	112.2 (68.29~184.2)	103.4 (55.21~193.8)
Delta + Alum		1150 (714.9~1851)	1002 (670.9~1495)	209.3 (106.5~411.3)	237.6 (153.9~367)

## Data Availability

Data are contained within the article.
